# Supplementary material for: In Vitro Antioxidant Properties and Phenolic Profile of Acid Aqueous Ethanol Extracts from Torreya grandis Seed Coat
Source: Molecules. 2022 Aug 29;27(17):5560. doi: 10.3390/molecules27175560 (PMC9457832; doi:10.3390/molecules27175560)
Supplement: Supplementary file 1 [file molecules-27-05560-s001.zip › molecules-1874932-supplementary.pdf]

Supplementary materials

In Vitro Antioxidant Properties and Phenolic Profile of Acid Aqueous Ethanol Extracts from *Torreya grandis* Seed Coat

Wei Quan [1,*], Yang Xu [1], Yiting Xie [1], Fei Peng [1] and Yong Lin [2,*]

[1] College of Food Science and Technology, Hunan Agricultural University, Changsha 410128, China
[2] Department of Food Science and Technology, Hunan Food and Drug Vocational College, Changsha 410128, China
[3] Engineering Technology for Utilization of Functional Ingredients from Botanicals, Hunan Agricultural University, Changsha 410128, China
* Correspondence: reus_quan@hunau.edu.cn (W.Q.); yong-lin@hunau.edu.cn (Y.L.); Tel./Fax: +86-510-85919065 (W.Q.)

Table S1. The total phenolic and flavonoid content of the ethanol extract from the *Torreya grandis* seed coat.

	**TPC**<br>**mg GA/g**	**TFC**<br>**mg RE/g**
TE	530.6 ± 20.7	25.5 ± 2.43

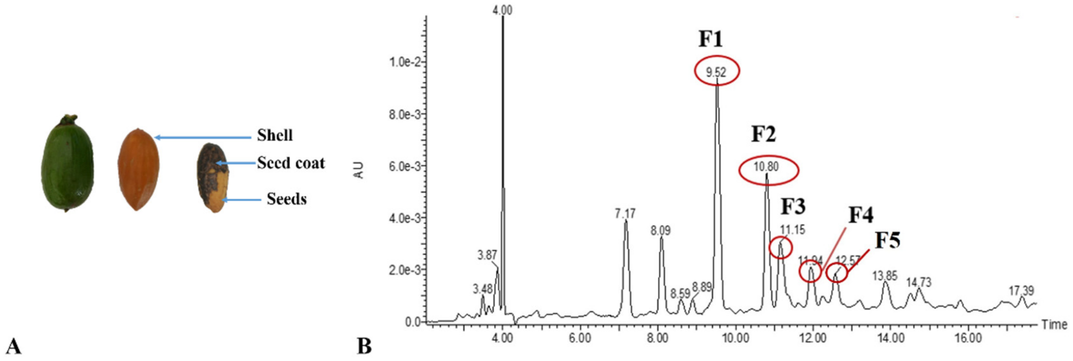

Figure S1. The picture of (**A**) *Torreya grandis* and *Torreya grandis* seeds and (**B**) HPLC-UV chromatogram of ethanolic extract from seed coat of Torreya grandis.

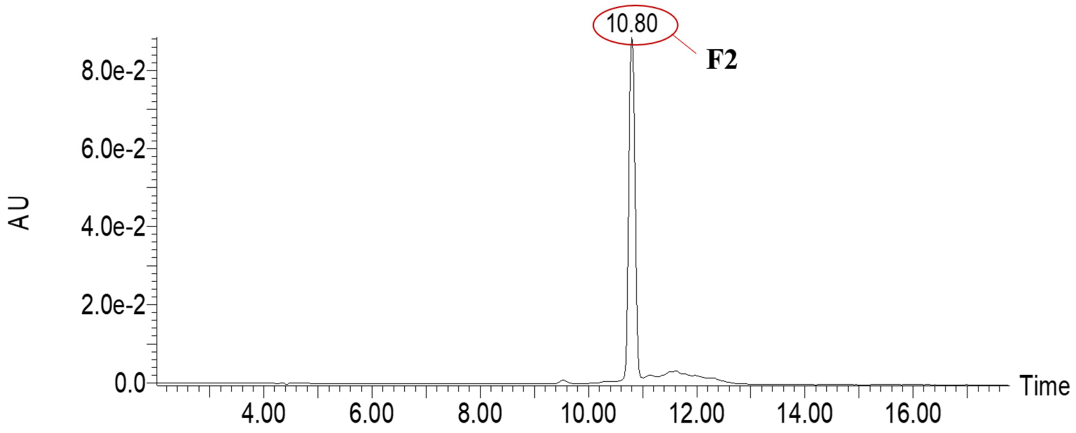

Figure S2. HPLC-UV chromatogram of peak F2 from seed coat of Torreya grandis.

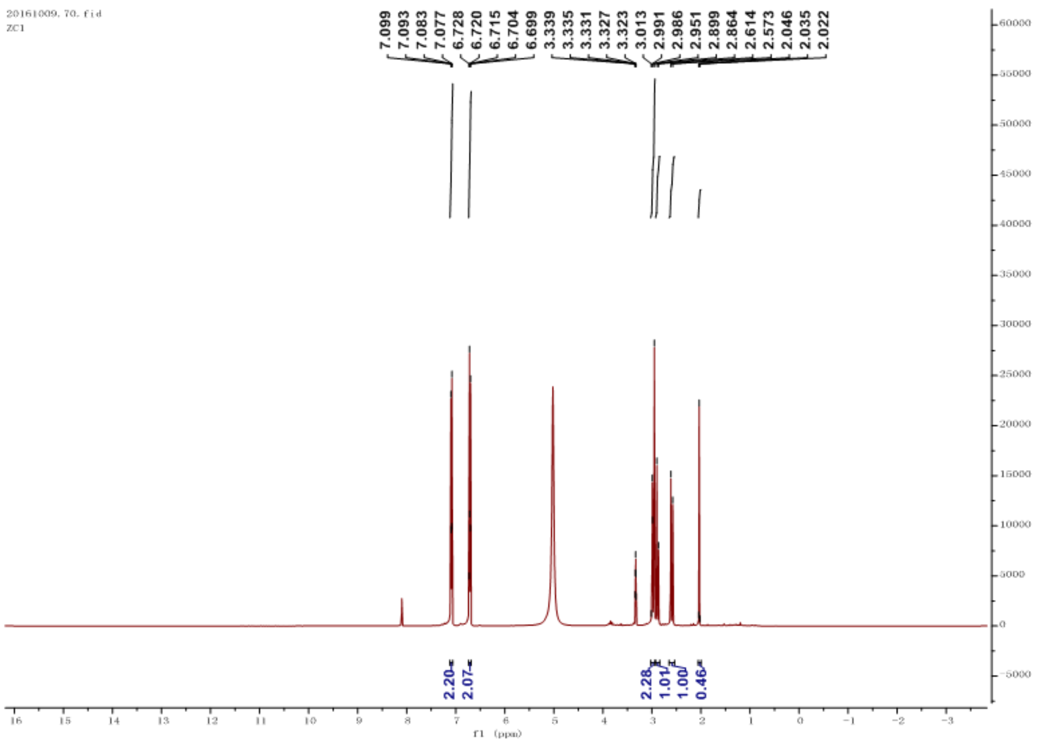

Figure S3. 1H NMR of 2-hydroxy-2-(4-hydroxyphenethyl) malonic acid.

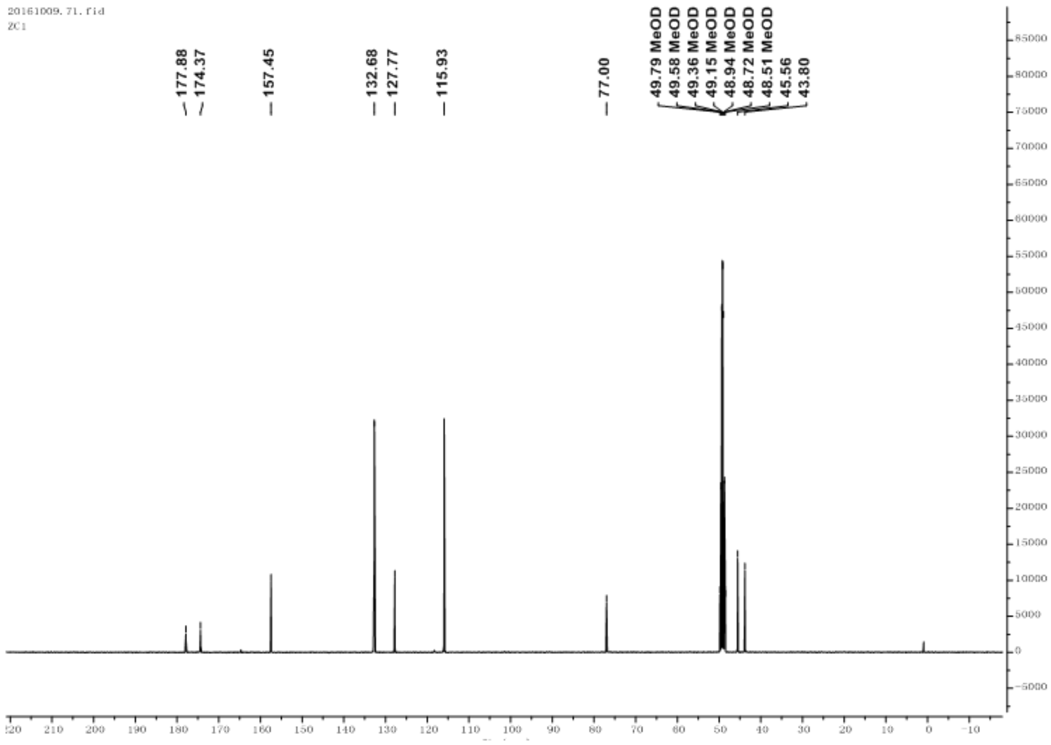

Figure S4. 13C NMR of 2-hydroxy-2-(4-hydroxyphenethyl) malonic acid.

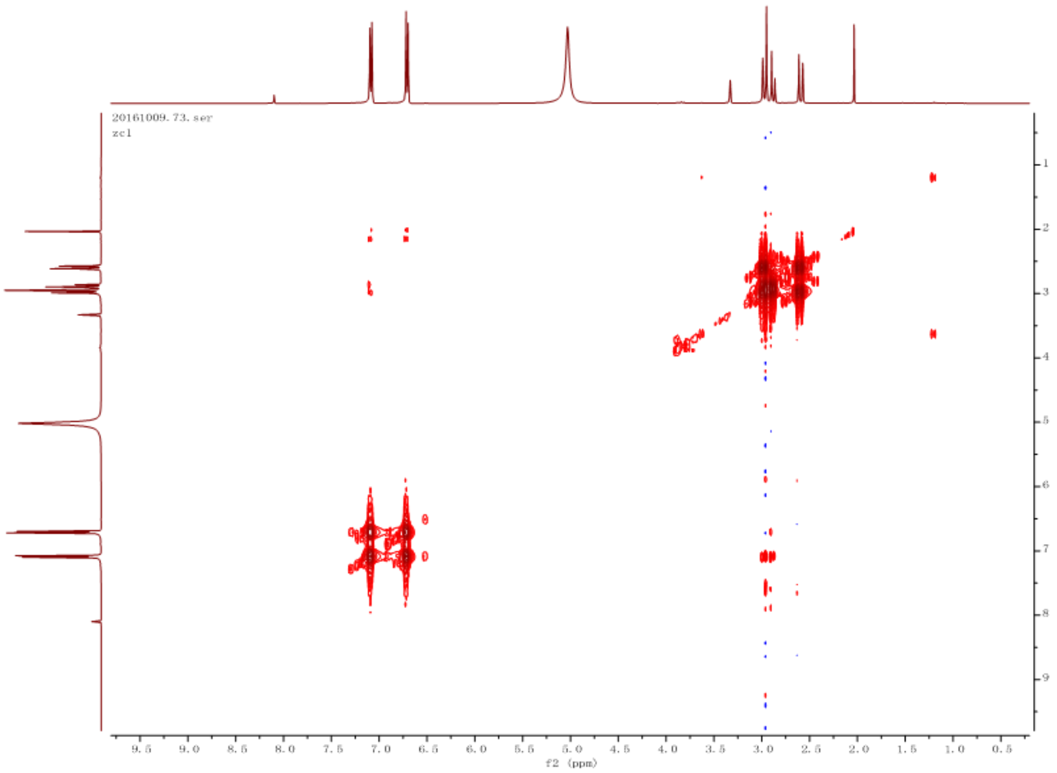

Figure S5. 1H-1H COSY of 2-hydroxy-2-(4-hydroxyphenethyl) malonic acid.

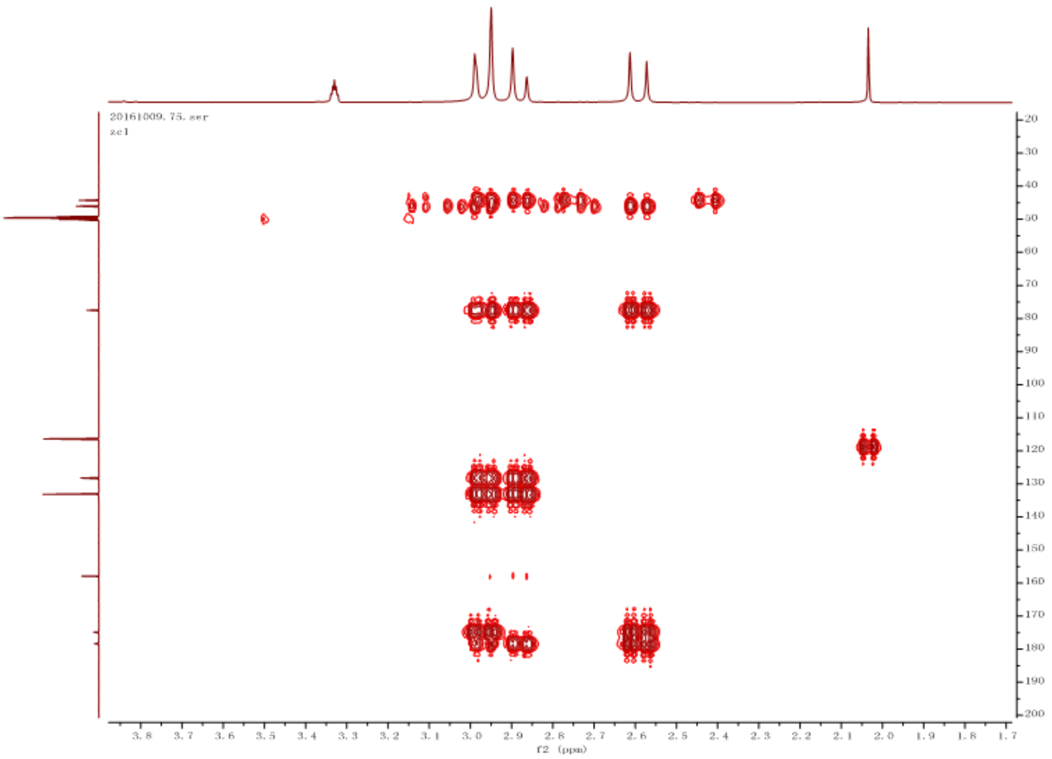

Figure S6. HMBC of 2-hydroxy-2-(4-hydroxyphenethyl) malonic acid.

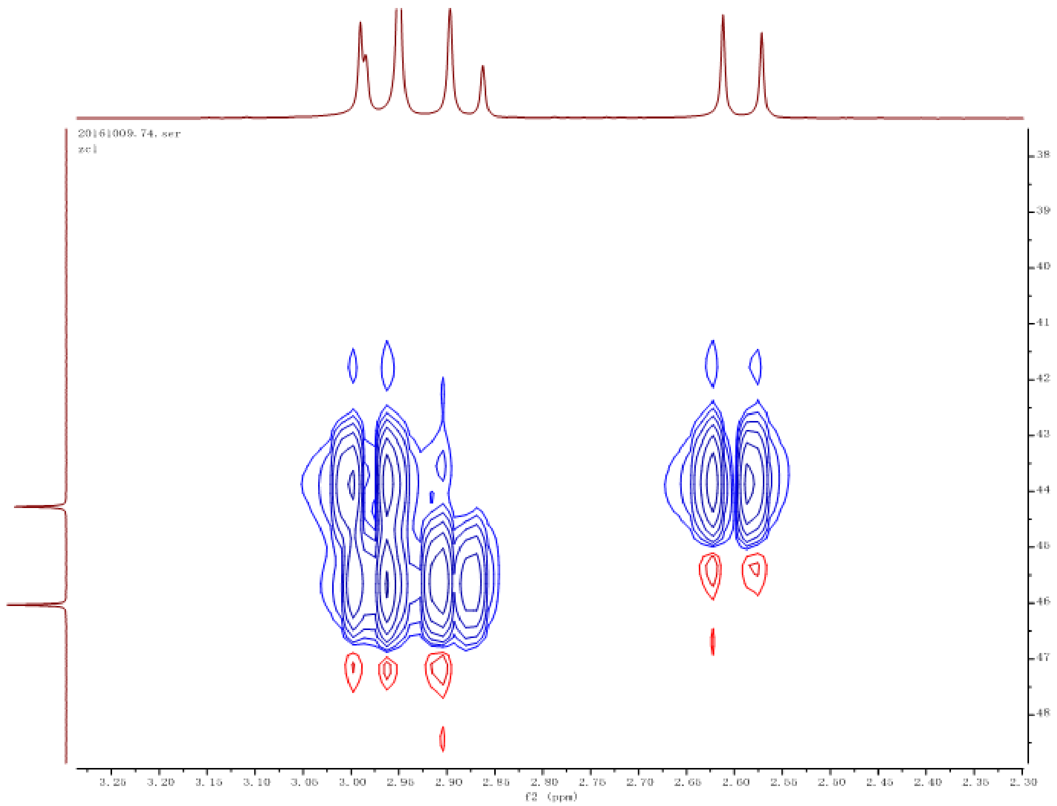

Figure S7. HSQC of 2-hydroxy-2-(4-hydroxyphenethyl) malonic acid.
